# Psychometric characteristics of the of COVID Stress Scales-Arabic version (CSS-Arabic) in Egyptian and Saudi university students

**DOI:** 10.1186/s43045-021-00095-8

**Published:** 2021-03-08

**Authors:** Adel S. Abbady, Abdel-Hady El-Gilany, Fathy A. El-Dabee, Adel M. Elsadek, Mahmoud ElWasify, Mohamed Elwasify

**Affiliations:** 1 College of Education, King Khalid University, Abha, Saudi Arabia; 2 College of Education, Aswan University, Aswan, Egypt; 3 Faculty of Medicine, Mansoura University, Mansoura, Egypt; 4 College of Education, Sohag University, Sohag, Egypt; 5 Psychiatry Department, Faculty of Medicine, Mansoura University, Mansoura, 35516 Egypt

**Keywords:** COVID-19, Stress, COVID stress scale, COVID stress scale-Arabic

## Abstract

**Background:**

Mental health sufferings due to the COVID-19 pandemic were reported in many countries worldwide. However, there is a lack of a validated Arabic tool to measure stress related to this pandemic in the Arab countries. This study aims to translate into Arabic and measure the psychometric characteristics of the previously developed English COVID Stress Scales (CSS). Using a forward-backward translation, the CSS was translated into Arabic and 22 jurors assessed its content validity. An online-based survey was carried out among 1080 university students (Egyptian and Saudi) to assess internal consistency and validity of the Arabic version (CSS-Arabic) using Cronbach’s *α* and factor analysis.

**Results:**

The content validity indices of the scale were 0.943 and 0.932 for both relevance and clarity. The internal consistency of the total CSS-Arabic was satisfactory (with *α* = 0.94) within the acceptable range for different subscales. Confirmatory factor analysis reveals 5-factor model with 36 retained items similar to the original English CSS.

**Conclusions:**

CSS-Arabic is a reliable and valid self-reporting tool for screening of stress due to COVID-19 among the university students. Further work should be done by healthcare providers to assess the magnitude of the stress during the COVID-19 pandemic.

**Supplementary Information:**

The online version contains supplementary material available at 10.1186/s43045-021-00095-8.

## Background

The newly emerging epidemic of the COVID-19 appeared at the end of 2019 in China and has been described as a pandemic by World Health Organization (WHO) [1]. The virus is transmitted to humans through small droplets that are sprinkled from the nose or mouth with coughing and sneezing or through small droplets scattered on the surfaces surrounding the human being. Its main symptoms are fever, dry cough, and fatigue, and some cases experience respiratory difficulties that necessitates intensive care [2].

This pandemic has drawn global attention largely focusing on the physical health as well as the clinical and diagnostic aspects. The psychological health effects cannot be overlooked [3]. With the widespread debate about the recently developed vaccine and empirically unproven effective treatments [4], countries were forced to adopt population-level widespread restrictions such as lock downs, in order to limit contact between people to slow down the transmission and exponential growth of cases [5].

The pandemic meets the criteria for a traumatic event and leads to the development of post-traumatic distress syndrome PTSD [6]. Stress affects one’s mental and physical well-being where the individual reflects the stressor and its stressfulness [7]. Stress is compounded by the alarming figures reported by local and international media all the time about the numbers of infections and deaths caused by the emerging COVID-19 virus; people are experiencing widespread panic, anxiety, and tension that humankind had not seen in a long period of time [8]. Many previous studies in different countries including Arabic ones revealed that the extent of the negative psychological effects of COVID-19 pandemic include stress, anxiety, despair, depression, suicidal thinking, sleep disorders, eating disorders, and decreased quality of life [9–17].

Taylor et al. [18] suggested psychological effects of COVID-19 underneath the name of COVID-19 stress syndrome, which is described by distress of becoming infected, distress of being contaminated, distress of foreigners who might be carrying infection (i.e., disease-related xenophobia), distress of the socio-economic consequences of the pandemic, compulsive checking and reassurance-seeking, and traumatic stress symptoms about the pandemic (e.g., nightmares, intrusive thoughts).

Taylor et al. [18] listed that healthcare suppliers should have a valid tool to evaluate the psychological effects of COVID-19 during the pandemic. To the best of the authors’ knowledge, the English SCC was not translated into other languages including Arabic.

The current study deals with the psychometric properties of the Arabized version from the stress of the COVID-19 pandemic and thus seeks to provide a scientifically sound measuring instrument based on the reality of Arab culture. Thus, standardization of the scale in the Arab environment opens new horizons for research in the field of diagnosing and measuring the stress associated with the pandemic of the COVID-19 and its psychological effects. This is an essential step in the confrontational strategies and psychotherapy practices. The ideal tool should be psychometrically tested for reliability and validity.

### Aim of the study

This study aims to translate into Arabic and measure the psychometric characteristics (content validity, internal consistency, and construct or component validity) of the COVID Stress Scales [18] in Egyptian and Saudi samples of university students.

## Methods

### Study design and date

This cross-section validation study was conducted during the period from 10th July to 23rd August 2020.

### Subjects

The study was carried out on university students in Mansoura University, Egypt, and King Khalid University, Saudi Arabia.

### Sample size calculation

There are no absolute rules for the sample size required to validate constructs or study tools due to the various types of tools and the number of their items [19]. Guidelines for the respondent-to-item ratio ranged from 5:1, 10:1, 15:1, or 30:1 [20]. As larger samples are always better than smaller samples, we will use the respondent-to-item ratio of 30:1 for better confirmatory factor analysis [19]. The scale contains 36 items; thus, the final sample size is 1080 students.

### Procedures and data collection

Participants were volunteered to enroll using an anonymous online survey, administered by Google Forms to ensure wide reach and easy access. The electronic link was published on social media and student forums; the participants agreed to participate voluntarily without any compensation or incentives. Data were collected anonymously including sociodemographics (e.g., age, sex, nationality, residence, and college) and the Arabic version of COVID-19 stress scale.

The COVID-19 Stress Scales (CSS) prepared by Taylor et al. [18] consists of 36 items, distributed over five dimensions stable factor, corresponding to scales assessing COVID-related stress and anxiety symptoms: (1) danger and contamination fears (12 items), (2) fears about economic consequences (6 items), (3) xenophobia (6 items), (4) compulsive checking and reassurance seeking (6 items), and (5) traumatic stress symptoms about COVID-19 (6 items). Items were rated on a 5-point scale ranging from 0 (not at all) to 4 (extremely). The scales were intentionally designed so they could be readily adapted for future pandemics. The CSS were developed and initially validated in population-representative samples from Canada and the USA. The scale performed well on various indices of reliability and validity. The scales were inter-correlated, providing evidence of a COVID stress syndrome.

### Arabic translation of CSS

The English version was translated into the Arabic language by two independent translators, and the two translations were compared to reach an agreed initial version. This initial version was revised from a linguistic, grammar, and other perspective. The Arabic version was back translated into the English language again by another two translators unaware about the original English version, to ensure the accuracy of the translation and the consistency of the synonyms and the translation did not differ between the English and Arabic versions [21].

### Content validity

The content validity indices were estimated by 22 experts (11 Egyptian and 11 Saudi) jurors specialized in psychology, psychiatry, and public health. They included 7 professors, 13 associate professors, and 2 lecturers. The Arabic version of the scale was evaluated for clarity, relevance, and translation of the contents. The experts were asked independently to review each item (clarity and relevance) using three-point ordinal scale and translation using correct or wrong. If wrong, they add their suggestions. The content validity index (CVI) was calculated at the item level (I-CVI), expert level (E-CVI), and scale level (S-CVI).

To obtain the CVI at the item level (I-CVI), the number of experts judging the item as relevant or clear (rating 3) were divided by the total number of experts. The CVI per expert (E-CVI) is calculated by dividing number of items scored 3/total number of items. If the CVI is higher than 0.79, the item was appropriate. If it is between 0.70 and 0.79, it needs revision. If it is less than 0.70, it is eliminated. The CVI for the entire scale (S-CVI) was assessed using the S-CVI with the average approach, by summing all I-CVI for relevancy divided by the number of items. The scale as a tool was considered to be valid if S-CVI greater than or equal to 0.90 [22].

I-CVI and S-CVI were calculated using the following formulae [23]:

$$ \mathrm{I}-\mathrm{CVI}=\mathrm{Number}\ \mathrm{of}\ \mathrm{experts}\ \mathrm{rating}\ \mathrm{the}\ \mathrm{item}\ 3/\mathrm{total}\ \mathrm{number}\ \mathrm{of}\ \mathrm{experts} $$

$$ \mathrm{S}-\mathrm{CVI}=\mathrm{Sum}\ \mathrm{of}\ \mathrm{the}\ \mathrm{I}-\mathrm{CVI}\mathrm{s}/\mathrm{total}\ \mathrm{number}\ \mathrm{of}\ \mathrm{items} $$

The CVI for each expert (E-CVI) is number of items scored 3 (relevant)/total number of items.

Reliability was assessed in the forms of internal consistency. To test the reliability of the CSS-Arabic, the final version was completed by1080 students. Internal consistency was examined by Cronbach’s *α* reliability coefficients. Cronbach’s *α* value of 0.50–0.70 was acceptable, whereas 0.70 or higher shows good homogeneity among the items [24].

### Exploratory factor analysis

Exploratory factor analysis of the 36 items was done using RML rotation using MPlus. Parallel analysis was used to determine the number of factors to retain. The selection of goodness-of-fit indices was based on conventional guidelines [25].

### Statistical analysis

Data were analyzed with SPSS version 24 (IBM Corporation, Chicago, IL, USA). Qualitative variables were presented as number and percent, whereas quantitative variables were presented as mean (SD). CVIs were calculated for each item and each expert. Cronbach’s *α* were calculated to measure the internal consistency between items. *P* ≤ 0.05 was considered statistically significant. Validity and factor analysis were determined using robust maximum likelihood (RML) rotation using MPlus examining the full scale [26]; the researchers used RML because it is robust to departures from a normality distribution of the data sample.

Internal consistency was assessed by Cronbach alpha coefficients (*α*), inter-item correlations and corrected item-total correlations. A Cronbach’s *α* of .70 or higher indicates acceptable reliability [27]. Goodness of fit was assessed according to the following criteria: root mean square error of approximation (RMSEA ≤ .10), comparative fit index (CFI > .90 or more desirably ≥ .95), and standardized root mean square residual (SRMR ≤ .08) [28, 29].

## Results

Table 1 shows that the I-CVI ranged from 0.818 to 1.0 in different items of both relevance and clarity. While the E-CVI ranged from 0.722 to 1.0 in different items of both relevance and clarity. The S-CVIs were 0.943 and 0.932 for relevance and clarity, respectively.

**Table 1:** Content validity indices (per item, per expert, and total scale) for both relevance and clarity of the SCC-Arabic

Item	I-CVI for relevance	I-CVI for clarity	Expert	E-CVI for relevance	E-CVI for clarity
1	0.909	1.0	1	1.0	1.0
2	0.909	1.0	2	0.917	0.861
3	0.864	0.909	3	0.975	0.833
4	0.818	0.955	4	1.0	0.972
5	0.955	0.955	5	0.944	0.917
6	0.909	0.864	6	1.0	1.0
7	0.955	1.0	7	0.975	1.0
8	0.909	0.955	8	0.972	1.0
9	0.818	1.0	9	1.0	1.0
10	0.864	0.955	10	1.0	1.0
11	0.909	1.0	11	1.0	1.0
12	0.818	0.955	12	0.972	1.0
13	1.0	0.909	13	0.722	0.722
14	1.0	0.864	14	0.972	1.0
15	1.0	0.909	15	0.944	1.0
16	0.818	0.864	16	0.944	0.975
17	0.955	0.909	17	.944	1.0
18	0.955	0.955	18	0.722	0.7.22
19	0.955	0.909	19	0.944	0.806
20	0.955	0.909	20	0.944	1.0
21	0.955	0.909	21	0.806	0.975
22	0.955	0.909	22	1.0	1.0
23	1.0	0.909			
24	0.909	0.909			
25	1.0	0.909			
26	1.0	0.818			
27	1.0	0.864			
28	0.955	0.909			
29	1.0	0.909			
30	1.0	0.909			
31	1.0	0.955			
32	1.0	0.955			
33	0.909	0.955			
34	1.0	1.0			
35	1.0	1.0			
36	1.0	0.955			
Total scale	0.943	0.932			

*CVI* Content validity index, *I* Item, *E* Expert

The mean age of participating students was 20.8 ± 1.9 years, 76.95 are females, 55% are Egyptians, 57.6% of rural residence, and 40.3% are enrolled arts and humanities faculties (Table 2).

**Table 2:** Sociodemographic characteristics of studied students (1080)

	*N* (%)
Age	
Mean (SD)	20.8 (1.9)
Median (minimum-maximum)	20.0 (17–36)
Sex	
Male	249 (23.1)
Female	831 (76.9)
Nationality	
Egypt	594 (55.0)
Saudi Arabia	486 (45.0)
Residence	
Urban	622 (57.6)
Rural	458 (42.4)
Faculty	
Medicine	306 (28.3)
Other health faculties^a^	175 (16.2)
Other practical^b^	164 (15.2)
Arts and humanities^c^	435 (40.3)

^a^Dentistry, pharmacy, nursing, and health sciences

^b^Veterinary, sciences, engineering, and agriculture

^c^Commerce, arts, laws, and education

Table 3 displays the exploratory factor analysis of the resulting of the 36 items. Good fit is indicated by SRMR (0.04) ≤ .08, RMSEA (0.07) ≤ .08, SRMR = 0.03 ≥ .95, and CFI (0.90) ≥ .90. The first five eigenvalues were as follows: 12.90, 2.97, 2.49, 1.67, and 1.31.

**Table 3:** Exploratory factor analysis and factor loadings of the SCC-Arabic

Item number	Scales	I	II	III	IV	V
1	DT	**0.56**	0.05	0.01	0.04	0.22
2	DT	**0.42**	0.04	0.02	0.06	0.17
3	DT	**0.52**	0.04	0.07	0.01	0.05
4	DT	**0.60**	0.03	0.02	0.05	0.03
5	DT	**0.42**	0.14	0.03	0.07	0.1
6	DT	**0.51**	0.11	0.07	0.02	0.01
7	DT	**0.73**	0.01	0.05	0.06	0.01
8	DT	**0.71**	0.03	0.00	0.14	0.11
9	DT	**0.62**	0.05	0.01	0.20	0.00
10	DT	**0.76**	0.02	0.03	0.10	0.03
11	DT	**0.61**	0.13	0.05	0.06	0.01
12	DT	**0.69**	0.19	0.10	0.17	0.09
13	SE	0.03	**0.74**	0.01	0.15	0.05
14	SE	0.06	**0.84**	0.03	0.04	0.00
15	SE	0.09	**0.76**	0.04	0.01	0.04
16	SE	0.09	**0.71**	0.03	0.14	0.15
17	SE	0.05	**0.77**	0.00	0.07	0.01
18	SE	0.06	**0.67**	0.00	0.11	0.21
19	X	0.03	0.18	**0.66**	0.00	0.04
20	X	0.05	0.04	**0.73**	0.05	0.01
21	X	0.01	0.05	**0.91**	0.05	0.00
22	X	0.03	0.04	**0.89**	0.01	0.04
23	X	0.16	0.02	**0.78**	0.10	0.03
24	X	0.10	0.15	**0.49**	0.28	0.11
25	T	0.17	0.02	0.10	**0.60**	0.01
26	T	0.24	0.01	0.00	**0.49**	0.13
27	T	0.06	0.04	0.01	**0.84**	0.05
28	T	0.13	0.01	0.02	**0.68**	0.12
29	T	0.02	0.06	0.02	**0.75**	0.03
30	T	0.01	0.01	0.01	**0.74**	0.11
31	CH	0.05	0.06	0.07	0.20	**0.67**
32	CH	0.04	0.08	0.09	0.04	**0.67**
33	CH	0.01	0.03	0.01	0.09	**0.73**
34	CH	0.01	0.07	0.09	0.17	**0.52**
35	CH	0.07	0.01	0.01	0.2	**0.63**
36	CH	0.06	0.03	0.10	0.00	**0.63**

Boldface indicates salient (> .30) loading

*DT* Danger and contamination, *SE* Socio-economic consequences, *X* Xenophobia, *T* Traumatic stress, *CH* Compulsive checking

**Fig. 1:**
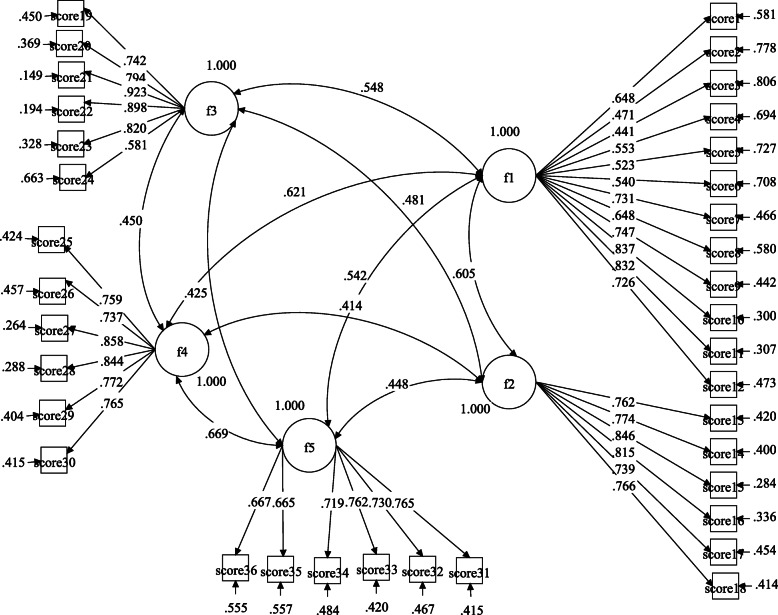
Five-factor implicit structure established with CFA by MPlus

**Fig. 2:**
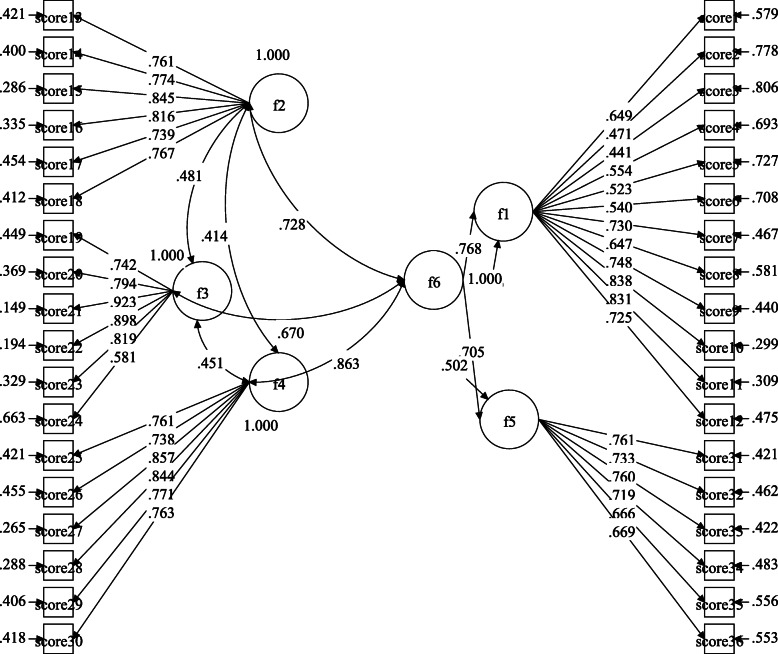
Second-order CFA by MPlus

Table 4 shows the first-order confirmatory factor analysis conducted for identifying the measurability of implicit structures of danger and contamination, socio-economic consequences, xenophobia, traumatic stress, and compulsive checking parameters used for CSS is depicted in Fig. 1. All of modification indication of the model have goodness of fit (*χ*^2^ = 2642.742, SRMR = 0.05, CFI = 0.91, TLI = 0.90, RMSEA = 0.07). Also, the second order confirmatory factor analysis for CSS indicating the structural of danger and contamination, socio-economic consequences, xenophobia, traumatic stress, and compulsive checking parameters dimensions with the one dimension of CSS is constructed and depicted in Fig. 2. All of modification indication of the model have goodness of fit (*χ*^2^ = 2681.171, SRMR = 0.05, CFI = 0.90, TLI = 0.90, RMSEA = 0.08).

**Table 4:** Findings of first and second order confirmatory factor analysis for CSS-Arabic

Measurement	*χ*^2^	df	*χ*^2^/df	SRMR	CFI	TLI	RMSEA
First-order confirmatory factor analysis							
CSS-Arabic	2642.742	584	4.525	0.05	0.91	0.90	0.07
Second-order confirmatory factor analysis							
CSS-Arabic	2681.171	586	4.575	0.05	0.90	0.90	0.08

*RMR* Root mean square residual, *CFI* Bentler’s Comparative Fit Index, *TLI* Tucker-Lewis index, *RMSEA* Root mean square error of approximation

Table 5 presents Cronbach alpha coefficients for the scales. The coefficients were > 0.80, indicating good to excellent.

**Table 5:** Cronbach alphas of CSS-Arabic

Subscales of CSS-Arabic	Cronbach alphas
COVID danger and contamination	0.90
COVID socioeconomic consequences	0.91
COVID xenophobia	0.90
COVID traumatic stress	0.91
COVID compulsive checking	0.86
Total CSS-Arabic	0.94

## Discussion

At the time of writing, the coronavirus pandemic is the most important health problem worldwide, with the starting of the second wave and emergence of new strains of the virus. Apart from its impacts on physical health, COVID-19 also has significant effects on mental health. The development of the vaccines does not alleviate the psychological effects of the COVID-19. Few tools are available for assessment of these psychological consequences in Arabic countries. The only available validated tools are the Arabic Fear of COVID-19 Scale (FCV-19S) [30] and the Arabic version of COVID-19 Anxiety Scale (CAS) [31]. CSS is the first tool that evaluates various aspects of psychological consequences of COVID related to five domains: COVID danger and contamination fears, COVID fears about economic consequences, COVID xenophobia, COVID compulsive checking and reassurance seeking, and COVID traumatic stress symptoms.

Content validity index (CVI) is the most commonly used method to calculate content validity quantitatively. There are multiple methods for testing content validity [32]. The most common method for measuring content validity is calculating the item level CVI (I-CVI). This paper considered the I-CVI, expert-level CVI (E-CVI), and scale CVI (S-CVI) since the S-CVI is an average score that can be skewed by outliers. It is important to distinguish between content validity at the item level and at the scale level [22].

The number of experts (*n* = 22) was considered adequate for content validation [33, 34]. An I-CVI of 0.78 or higher is considered excellent. It was concluded that individual items were important and relevant to measuring the content validity. The minimum acceptable S-CVI is considered to be any value between 0.80 and 0.90 [22, 34]. In the current study, the CVIs are satisfactory for relevance and clarity ranged from 0.8 to 1.0 for I-CVI, E-CVI, and S-CVI.

The current study revealed that the Cronbach alphas of the different domains are satisfactory and ranged from 0.86 to 0.91. These are comparable to those of the original English version of SCC. Cronbach alphas ranged from 0.83 to 0.94 in the Canadian sample and from 0.86 to 0.95 in the US sample [18].

Factor analysis revealed 5-factor loading. This analysis using the total score and the all five factors would likely yield the most clinically reliable information regarding COVID-19 stress severity, particularly when used in population-based samples. Exploratory factor analysis was done using RML rotation using MPlus; good fit is indicated by SRMR (0.05) ≤ .08, RMSEA (0.07) ≤ .08, TLI = 0.90 ≤ .90, and CFI (0.91) ≤ .90. Also, findings of first and second order confirmatory factor analysis for CSS resulted the 5-factor model from the sample was tested. CFA solution performed gives goodness of fit in modification indication.

The obtained results are in harmony with that detected by Taylor et al. [18] who demonstrated that parallel analysis indicated a 5-factor solution, rather than a 6-factor solution in which each factor corresponded to each of the six scales of the CSS. The first six Eigen values were as follows: 15.84, 2.86, 2.06, 1.58, 1.55, and 0.88. The model performed well in terms of the goodness-of-fit and indicates RMSEA = 0.050 (90% confidence interval 0.049−0.051), SRMR = 0.042, and CFI = .93.

The results of the SCC-Arabic’s internal consistency revealed a good to excellent Cronbach’s *α* coefficient. The alphas of the different domains ranged from 0.86 to 0.91 with a total scale coefficient of 0.94. These findings are comparable to coefficients of the original English scale (with *α* ranging from 0.80 to 0.93 for different domains) in both Canadian and US populations [18].

### Limitations

First, the study was conducted using online modalities, which might make the measure inaccessible to some individuals. However, given the requirement of quarantine and physical distancing, online surveys might be the only option during certain periods of a pandemic. This may have introduced selection bias, where only those who received the study link had the chance to participate.

Second, participants were recruited only from university students who have accessibility to internet. Also, they were not representative of the adult population in both countries. Future studies involving more diverse adult samples from different countries would help further establish the validity and utility of CSS-Arabic. Third, test-retest and inter-rater reliabilities as well as scale sensitivity and specificity were not examined for the SCC-Arabic. Finally, there was no evidence of convergent or discriminant validity.

## Conclusions

The findings show that the Arabic CSS had a multidimensional structure, good internal consistency, and good construct validity. The authors believe that the CSS-Arabic is a potentially useful instrument that can be used by healthcare providers to assess the magnitude of the stress during the COVID-19 pandemic. It may provide comprehensive information regarding stress and anxiety of an individual because of its multidimensional structure. However, the CSS is long and takes a longer time to complete.

## Supplementary Information

**Additional file 1.** Arabic CSS.

**Additional file 2.**

**Additional file 3.**

## Data Availability

Data are available upon request from the corresponding author. Also, CSS-Arabic is available as an Additional file 1.

## Abbreviations

COVID-19: Coronavirus disease
CSS: COVID Stress Scales
CVI: Content validity index
I-CVI: Item content validity index
E-CVI: Expert content validity index
DT: Danger and contamination
SE: Socio-economic consequences
X: Xenophobia
T: Traumatic stress
CH: Compulsive checking
RMR: Root mean square residual
CFI: Bentler’s Comparative Fit Index
TLI: Tucker-Lewis index
RMSEA: Root mean square error of approximation
