# Supplementary material for: Psychometric characteristics of the of COVID Stress Scales-Arabic version (CSS-Arabic) in Egyptian and Saudi university students
Source: Middle East Curr Psychiatry. 2021 Mar 8;28(1):14. doi: 10.1186/s43045-021-00095-8 (PMC7938384; doi:10.1186/s43045-021-00095-8)
Supplement: Supplementary file 1 — Additional file 1. Arabic CSS. [file 43045_2021_95_MOESM1_ESM.docx]

**مقياس ضغوط جائحة كورنا المستجد (كوفيد-19)**

**فيما يلي مجموعة من العبارات عن أنواع مختلفة من المخاوف التي ربما تكون قد عانيت منها خلال الأيام السبعة الماضية.**

**ملاحظة:**

- **نشير كلمة "الفيروس" إلى فيروس كورنا المستجد (كوفيد-19).**
- **تشير "العزلة الذاتية" إلى الانفصال الطوعي عن الآخرين.**

| م | العــبارة | موافق بشدة | موافق | محايد | غير موافق | | غير موافق بشدة | |
| --- | --- | --- | --- | --- | --- | --- | --- | --- |
| 1 | تقلقني إصابتي بالفيروس. |  |  |  |  | |  | |
| 2 | يقلقني عدم قدرتي على حماية عائلتي من الإصابة بالفيروس. |  |  |  |  | |  | |
| 3 | يقلقني عدم قدرة نظام الرعاية الصحية لدينا على حماية أحبائي من الفيروس. |  |  |  |  | |  | |
| 4 | يقلقني عدم قدرة نظام الرعاية الصحية لدينا على حمايتي من الفيروس. |  |  |  |  | |  | |
| 5 | يقلقني عدم كفاية اتباع العادات الصحية الأساسية (كغسل اليدين) لحمايتي من الفيروس. |  |  |  |  | |  | |
| 6 | يقلقني أن التباعد الاجتماعي ليس كافيًا لحمايتي من الفيروس. |  |  |  |  | |  | |
| 7 | يقلقني أن لمسي لشيء ما في مكان عام (درابزين، مقبض باب)، سوف يصيبني بالفيروس. |  |  |  |  | |  | |
| 8 | يقلقني أن سعال شخص ما أو عطسه بالقرب مني، سوف يصيبني بالفيروس. |  |  |  |  | |  | |
| 9 | يقلقني أن الأشخاص المحيطين بي سيصيبونني بالفيروس. |  |  |  |  | |  | |
| 10 | يقلقني أخذ باقي النقود في المعاملات النقدية. |  |  |  |  | |  | |
| 11 | يقلقني أنني قد أصاب بالفيروس من التعامل بالنقود أو استخدام آلة الصراف الآلي. |  |  |  |  | |  | |
| 12 | يقلقني تلوث بريدي بالفيروس بواسطة ساعي البريد. |  |  |  |  | |  | |
| 13 | يقلقني نفاد المواد التموينية من محلات البقالة. |  |  |  |  | |  | |
| 14 | يقلقني إغلاق متاجر البقالة. |  |  |  |  | |  | |
| 15 | يقلقني نفاد مواد التنظيف أو المطهرات من الأسواق. |  |  |  |  | |  | |
| 16 | يقلقني نفاد علاجات البرد أو الإنفلونزا من الأسواق. |  |  |  |  | |  | |
| 17 | يقلقني نفاد المياه من محلات البقالة. |  |  |  |  | |  | |
| 18 | يقلقني نفاد الأدوية الموصوفة من الصيدليات. |  |  |  |  | |  | |
| 19 | يقلقني أن الأجانب قد ينشرون الفيروس في بلدي. |  |  |  | |  | |  |
| 20 | إذا ذهبت إلى مطعم متخصص في الأطعمة الأجنبية، سأكون قلقًا بشأن الإصابة بالفيروس. |  |  |  | |  | |  |
| 21 | يقلقني الاختلاط بالأجانب لأنهم قد يكونون حاملين للفيروس. |  |  |  | |  | |  |
| 22 | إذا قابلت شخصًا من بلد أجنبي، سأكون قلقًا من احتمال إصابته بالفيروس. |  |  |  | |  | |  |
| 23 | إذا كنت في مصعد مع مجموعة من الأجانب، سأكون قلقًا من أنهم مصابون بالفيروس. |  |  |  | |  | |  |
| 24 | يقلقني أن الأجانب قد ينشرون الفيروس لأنهم ليسوا بمستوى نظافتنا. |  |  |  | |  | |  |

**يرجى قراءة كل عبارة من العبارات التالية، والإشارة إلى مدى المرات التي واجهت فيها كل مشكلة خلال الأيام السبعة الماضية.**

|  | | أبداً | نادراً | أحياناً | غالبًا | دائماً |
| --- | --- | --- | --- | --- | --- | --- |
| 25 | واجهت صعوبة في التركيز لأنني انشغلت بالتفكير في الفيروس. |  |  |  |  |  |
| 26 | جالتْ بخاطري صور ذهنية مزعجة حول الفيروس رغمًا عني. |  |  |  |  |  |
| 27 | واجهت صعوبة في النوم نتيجة قلقي من الفيروس. |  |  |  |  |  |
| 28 | أفكر في الفيروس على الرغم من أنني لا أقصد ذلك. |  |  |  |  |  |
| 29 | تذكري الفيروس يسبب لي ردود فعل جسدية، مثل التعرق أو سرعة ضربات القلب. |  |  |  |  |  |
| 30 | راودتنى أحلام سيئة حول الفيروس. |  |  |  |  |  |

**العبارات التالية تهدف الي التحقق من مقدار السلوكيات التي قمت بسبب المخاوف بشأن فيروس كورنا المستجد (كوفيد19) خلال الأيام السبعة الماضية؟**

|  | | أبداً | نادراً | أحياناً | غالبًا | دائماً |
| --- | --- | --- | --- | --- | --- | --- |
| 31 | أبحث في الإنترنت عن علاجات الفيروس |  |  |  |  |  |
| 32 | اطلب المشورة من المهنيين الصحيين (الأطباء أو الصيادلة) بشأن الفيروس |  |  |  |  |  |
| 33 | أشاهد مقاطع فيديو حول الفيروس. |  |  |  |  |  |
| 34 | أفحص جسدي بحثًا عن علامات العدوى بالفيروس. |  |  |  |  |  |
| 35 | أسعى للاطمئنان من الأصدقاء أو العائلة حول الفيروس. |  |  |  |  |  |
| 36 | أطلع على منشورات وسائل التواصل الاجتماعي بشأن الفيروس. |  |  |  |  |  |

**البعد الأول: المخاطر والتلوث بـكوفيد (19): العبارات من (1-12)**

**البعد الثاني: العواقب الاجتماعية الاقتصادية لـكوفيد (19):** **العبارات من (13-18)**

**البعد الثالث: رهاب (خوف) الأجانب الراجع لكوفيد (19):** **العبارات من (19-24)**

**البعد الرابع: أعراض الضغوط الصادمة لـكوفيد (19):** **العبارات من (25-30)**

**البعد الخامس: الفحص القهري بسبب كوفيد (19):** **العبارات من (31-36)**
