# Supplementary material for: Psychometric characteristics of the of COVID Stress Scales-Arabic version (CSS-Arabic) in Egyptian and Saudi university students
Source: Middle East Curr Psychiatry. 2021 Mar 8;28(1):14. doi: 10.1186/s43045-021-00095-8 (PMC7938384; doi:10.1186/s43045-021-00095-8)
Supplement: Supplementary file 2 — Additional file 2. [file 43045_2021_95_MOESM2_ESM.pdf]

adel.sayed

From: Gordon Asmundson <gordon.asmundson@uregina.ca>
Sent: Monday, June 1, 2020 9:50 PM
To: adel.sayed
Cc: Caeleigh Landry
Subject: Re: the COVID Stress Scales

Dear Adel:

Thank you again for your request. We are granting you permission to translate the COVOD Stress Scales (CSS) into Arabic. We are not granting permission to modify the scales (i.e., to make shortened versions). We have attached a PDF of the CSS as well as scoring template here for your use.

Sincerely,

Dr. Asmundson

****
Gordon J. G. Asmundson, Ph.D., R. D. Psych
Fellow of Royal Society of Canada
CACBT Certified in Cognitive Behaviour Therapy
Professor of Psychology
Co-Director, PsyPAN Network (coronaphobia.org)

Editor-in-Chief, Journal of Anxiety Disorders
http://www.journals.elsevier.com/journal-of-anxiety-disorders

Development Editor, Clinical Psychology Review
https://www.journals.elsevier.com/clinical-psychology-review/

University of Regina
3737 Wascana Parkway
Regina, Saskatchewan
CANADA S4S 0A2

access articles at

https://scholar.google.ca/scholar?hl=en&as_sdt=0%2C5&q=gordon+asmundson&oq=

web: aibl.ca
phone: (306) 337-2415
fax: (306) 337-3275
e-mail: gordon.asmundson@uregina.ca
****

On May 30, 2020, at 3:28 PM, adel.sayed <adel_abbady@aswu.edu.eg> wrote:

Thank you for your interest in responding, I intend to Standardization the scale in the Kingdom of Saudi Arabia, Prof Fathi El-Dabee participates in the research, from College of Education- Sohag University, and We are now working in the Kingdom of Saudi Arabia at King Khalid University.
If you are interested, you can participate with us in the research, and it will be an addition to your biography.

From: Gordon Asmundson <gordon.asmundson@uregina.ca>
Sent: Wednesday, May 27, 2020 5:46 PM
To: adel.sayed <adel_abbady@aswu.edu.eg>
Subject: Re: the COVID Stress Scales

Thank you for your request. Can you provide some additional details regarding your planned translation and study and who will be involved? We have been receiving multiple requests for translations into various languages and are seriously considering all requests.

Sincerely,

Dr. Asmundson

****
Gordon J. G. Asmundson, Ph.D., R. D. Psych
Fellow of Royal Society of Canada
CACBT Certified in Cognitive Behaviour Therapy
Professor of Psychology
Co-Director, PsyPAN Network (coronaphobia.org)

Editor-in-Chief, Journal of Anxiety Disorders
http://www.journals.elsevier.com/journal-of-anxiety-disorders

Development Editor, Clinical Psychology Review
https://www.journals.elsevier.com/clinical-psychology-review/

University of Regina
3737 Wascana Parkway
Regina, Saskatchewan
CANADA S4S 0A2

access articles at

https://scholar.google.ca/scholar?hl=en&as_sdt=0%2C5&q=gordon+asmundson&oq=

web: aibl.ca
phone: (306) 337-2415
fax: (306) 337-3275
e-mail: gordon.asmundson@uregina.ca
****

On May 25, 2020, at 5:02 PM, adel.sayed <adel_abbady@aswu.edu.eg> wrote:

Prof, Taylor, S.

I wish to be in good health, and I hope to have your agree to translate your scale about the COVID Stress Scales in your paper "Development and initial validation of the COVID Stress Scales" to Arabic language in my research. Kindly send me an email to acknowledge the agree of this mail.
Your student

Abbady, A

Mental Health Dept.,

Faculty of Education,

Aswan University, Aswan, Egypt.
