# Supplementary material for: Psychometric characteristics of the of COVID Stress Scales-Arabic version (CSS-Arabic) in Egyptian and Saudi university students
Source: Middle East Curr Psychiatry. 2021 Mar 8;28(1):14. doi: 10.1186/s43045-021-00095-8 (PMC7938384; doi:10.1186/s43045-021-00095-8)
Supplement: Supplementary file 3 — Additional file 3. [file 43045_2021_95_MOESM3_ESM.pdf]

The COVID Stress Scales

The following asks about various kinds of worries that you might have experienced ***over the past seven days***. In the following statements, we refer to COVID-19 as "the virus".

Please note: "**self-isolation**" refers to ***voluntary*** separation from others.

	Not at all	Slightly	Moderately	Very	Extremely
**1. I am worried about catching the virus**	0	1	2	3	4
**2. I am worried that I can't keep my family safe from the virus**	0	1	2	3	4
**3. I am worried that our healthcare system won't be able to protect my loved ones**	0	1	2	3	4
**4. I am worried our healthcare system is unable to keep me safe from the virus**	0	1	2	3	4
**5. I am worried that basic hygiene (e.g., handwashing) is not enough to keep me safe from the virus**	0	1	2	3	4
**6. I am worried that social distancing is not enough to keep me safe from the virus**	0	1	2	3	4
**7. I am worried about grocery stores running out of food**	0	1	2	3	4
**8. I am worried that grocery stores will close down**	0	1	2	3	4
**9. I am worried about grocery stores running out of cleaning or disinfectant supplies**	0	1	2	3	4
**10. I am worried about grocery stores running out of cold or flu remedies**	0	1	2	3	4
**11. I am worried about grocery stores running out of water**	0	1	2	3	4
**12. I am worried about pharmacies running out of prescription medicines**	0	1	2	3	4
**13. I am worried that foreigners are spreading the virus in my country**	0	1	2	3	4
**14. If I went to a restaurant that specialized in foreign foods, I'd be worried about catching the virus**	0	1	2	3	4

**15. I am worried about coming into contact with foreigners because they might have the virus**	0	1	2	3	4
**16. If I met a person from a foreign country, I'd be worried that they might have the virus**	0	1	2	3	4
**17. If I was in an elevator with a group of foreigners, I'd be worried that they're infected with the virus**	0	1	2	3	4
**18. I am worried that foreigners are spreading the virus because they're not as clean as we are**	0	1	2	3	4
**19. I am worried that if I touched something in a public space (e.g., handrail, door handle), I would catch the virus**	0	1	2	3	4
**20. I am worried that if someone coughed or sneezed near me, I would catch the virus**	0	1	2	3	4
**21. I am worried that people around me will infect me with the virus**	0	1	2	3	4
**22. I am worried about taking change in cash transactions**	0	1	2	3	4
**23. I am worried that I might catch the virus from handling money or using a debit machine**	0	1	2	3	4
**24. I am worried that my mail has been contaminated by mail handlers**	0	1	2	3	4

Please read each statement and indicate how frequently you have experienced each problem ***during the past seven days.***

	**Never**	**Rarely**	**Sometimes**	**Often**	**Almost Always**
**25. I had trouble concentrating because I kept thinking about the virus**	0	1	2	3	4
**26. Disturbing mental images about the virus popped into my mind against my will**	0	1	2	3	4
**27. I had trouble sleeping because I worried about the virus**	0	1	2	3	4
**28. I thought about the virus when I didn't mean to**	0	1	2	3	4

**29. Reminders of the virus caused me to have physical reactions, such as sweating or a pounding heart**	0	1	2	3	4
**30. I had bad dreams about the virus**	0	1	2	3	4

The following items ask about checking behaviours. ***<u>During the past seven days,</u>*** how much have you done the following because of concerns about COVID-19?

	**Never**	**Rarely**	**Sometimes**	**Often**	**Almost Always**
**31. Searched the Internet for treatments for COVID-19**	0	1	2	3	4
**32. Asked health professionals (e.g., doctors or pharmacists) for advice about COVID-19**	0	1	2	3	4
**33. Checked YouTube videos about COVID-19**	0	1	2	3	4
**34. Checked your own body for signs of infection (e.g., taking your temperature)**	0	1	2	3	4
**35. Sought reassurance from friends or family about COVID-19**	0	1	2	3	4
**36. Checked social media posts concerning COVID-19**	0	1	2	3	4

Found in: Taylor, S., Landry, C. A., Paluszek, M. M., Fergus, T. A., McKay, D. & Asmundson, G. J. G. (2020). Development and initial validation of the COVID Stress Scales. Journal of Anxiety Disorders. doi.org/10.1016/j.janxdis.2020.102232

Scoring:

Danger subscale:	Add scores for items 1-6	__________
Socio-economic consequences subscale:	Add scores for items 7-12	__________
Xenophobia subscale:	Add scores for items 13-18	__________
Contamination subscale:	Add scores for items 19-24	__________
Traumatic Stress subscale:	Add scores for items 25-30	__________
Compulsive Checking subscale:	Add scores for items 31-36	__________
Total:		__________
